# Artificial Gravity Reveals that Economy of Action Determines the Stability of Sensorimotor Coordination

**DOI:** 10.1371/journal.pone.0005248

**Published:** 2009-04-13

**Authors:** Richard G. Carson, Yalchin Oytam, Stephan Riek

**Affiliations:** 1 School of Psychology, Queen's University Belfast, Belfast, United Kingdom; 2 Perception and Motor Systems Laboratory, University of Queensland, Queensland, Australia; 3 CSIRO Molecular & Health Technologies, New South Wales, Australia; Victoria University of Wellington, New Zealand

## Abstract

**Background:**

When we move along in time with a piece of music, we synchronise the downward phase of our gesture with the beat. While it is easy to demonstrate this tendency, there is considerable debate as to its neural origins. It may have a structural basis, whereby the gravitational field acts as an orientation reference that biases the formulation of motor commands. Alternatively, it may be functional, and related to the economy with which motion assisted by gravity can be generated by the motor system.

**Methodology/Principal Findings:**

We used a robotic system to generate a mathematical model of the gravitational forces acting upon the hand, and then to reverse the effect of gravity, and invert the weight of the limb. In these circumstances, patterns of coordination in which the upward phase of rhythmic hand movements coincided with the beat of a metronome were more stable than those in which downward movements were made on the beat. When a normal gravitational force was present, movements made down-on-the-beat were more stable than those made up-on-the-beat.

**Conclusions/Significance:**

The ubiquitous tendency to make a downward movement on a musical beat arises not from the perception of gravity, but as a result of the economy of action that derives from its exploitation.

## Introduction

We are all familiar with the experience of tapping in time with a well-known tune. Invariably we will coordinate our movements so that the end of the downward phase of our gesture coincides with the beat of the music. It is widely held that spontaneous coordination phenomena of this kind have a structural basis [1], [2], and that a cognitive representation of perceived events and planned actions in a common coding scheme is integral to sensorimotor synchronisation [3]. In this regard, it has been proposed that the perception of invariant constraints in the environment, of which the direction of gravity is the most fundamental, plays a critical role [4]. It is evident that the currently experienced gravitational force does not define the perceived gravitational reference frame. In orbital spaceflight, in the absence of gravity-determined sensory (i.e. otolith) or body orientation cues, astronauts inappropriately apply a model of Earth's gravity when reaching to catch balls that are directed downwards from the ceiling to the floor of a space module [5]. Thus, the CNS does not treat gravitational force simply as an external load, but rather as an orientation reference that biases the formulation of motor commands [6]. The implication is that the tendency to use downward movement as the basis for synchronisation, and the greater precision and accuracy in timing that ensues from moving down on the beat reflects an embedded cognitive representation of the direction of gravity.

An alternative view is that precedence of downward motion arises from a functional exploitation of the gravitational forces. In this scheme the key factor determining the preferred strategy of synchronisation (moving down on the beat) is the greater economy with which the phase of the movement assisted by gravity can be performed by the motor system. In the present study we sought to assess the relative merits of the two explanations by temporarily uncoupling the direction of the gravitational force from the orientation indicated by the environment. If the tendency to move down on the beat stems from a structural internalisation of the orientation of terrestrial gravity, then temporarily altering the direction of the gravitational force should have no or minimal effect on coordination. If, on the other hand, it stems from the downward movement being assisted by the gravitational force, then the reversal of the gravitational force should significantly influence the stability of synchronisation. In its strongest sense, this hypothesis would predict that synchronisation would reverse (to moving up on the beat) with the reversal of the gravitational force.

Naïve volunteers were asked to rhythmically raise and lower their hand in time with a metronome. To dissociate the direction of motion from the muscle force necessary to move the hand with respect to gravity, we introduced a unique procedure to invert the weight of the hand. A robotic system was used to generate a mathematical model of the gravitational forces acting upon each participant's hand as it rotated around the wrist joint. By deriving the mathematical inverse of this model, we were then able to use the robot to apply torques that compensated completely for the weight of the hand. In separate blocks of trials we either re-introduced the normal gravitational torque, such that if a participant was asked to fully relax their muscles the hand fell downward, or applied a torque of the same magnitude in the opposite direction such that when completely relaxed the hand would rise upward. On each trial, the participants were instructed to either move “up-on-the-beat” or “down-on-the-beat” of the metronome. In order to challenge the stability of coordination, we increased the frequency of the metronome continuously throughout each trial. Performance was assessed in terms of the ability of the participants to maintain the initially prescribed pattern of coordination. The outcome was unambiguous - the most stable pattern of coordination was always that in which the phase of the movement that was assisted by the weight of the limb was synchronised with the beat, regardless of whether that phase was up or down.

## Methods

### Participants and Apparatus

The experiments included eight male and female volunteers (21–35 years), with no known neurological or movement deficits. All gave written informed consent to the procedures, which were approved by the University of Queensland Medical Ethics Committee and conducted in accordance with the Declaration of Helsinki. They were seated in a comfortable chair. In some trials the forearms were supported and stabilised in a prone position, in others the forearms were placed in a supine position. The elbows were semi-flexed (100° to 120°), and the upper arms restrained against the torso. The right hand was fixated at mid palm in a manipulandum, coupled via a shaft located coaxially with the axis of rotation of the wrist to a robotic system comprising an AC servo motor (Baldor BSM 4250AA, Fort Smith, AR, U.S.A.), controlled via a real-time digital signal processing interface (dSPACE, Paderborn, Germany). The metronome consisted of a 50 ms sine wave (540 Hz) tone generated by a microcomputer. Intervals between successive beats of the metronome were decreased progressively from 1.50 to 3.50 Hz over 50 s, as described by a chirp function. The electromyographic (EMG) activity of flexor carpi radialis (FCR), and extensor carpi radialis (ECR) longus of the right arm was recorded using bipolar surface electrodes. EMG signals were amplified and bandpass (30 Hz–1 kHz) filtered, digitised, and sampled along with the transduced position of the hand at 2 kHz.

### Generation of Inverse Models

We applied a 0–3.5 Hz bandwidth stochastic signal as torque input to the motor and measured the resultant angular velocity of the manipulandum. The Box-Jenkins method was used to establish a mathematical model of the motor as the best linear fit between the input (torque) and output (angular velocity) signals. The inverse of this model was then used as a controller to cancel the inertial characteristics of the motor. This method was then applied for each participant, separately for the prone and supine forearm positions, to identify and compensate for the unique dynamics of their hand. During the modelling process, audio feedback of EMG activity was provided to the participants to ensure that they remained passive.

By implementing these system identification and inverse control techniques, we were able to use the motor to apply an ideal source of torque to the limb without otherwise interfering with the dynamics of the limb. In the normal weight condition, the customary gravitational torque acting upon the limb was re-introduced. In the inverted weight condition, a torque of precisely the same magnitude was applied in the opposite direction. In the inverted weight condition therefore, the hand was subject to a torque applied at the wrist that caused it to rise upwards, as if subject to reverse gravity. In the normal weight condition the hand was subject to a torque that caused it to fall downwards, as per real gravity.

### Task

Individuals raised and lowered their hand in time with the metronome, commencing each trial in one of the two prescribed patterns of coordination: moving up-on-the-beat or moving down-on-the-beat. When the forearm was in a prone position, moving down-on-the-beat required that a wrist flexion movement coincide with the beat of the metronome, whereas moving up-on-the-beat required that a wrist extension movement coincide with the beat of the metronome. When the forearm was in a supine position, moving down-on-the-beat required that a wrist extension movement coincide with the beat of the metronome, whereas moving up-on-the-beat required that a wrist flexion movement coincide with the beat of the metronome. The participants were required to produce one full cycle of movement for each beat of the metronome. They were instructed to maintain the initial pattern as accurately as possible as the frequency of the metronome increased, but were also told that should the pattern change they were not to intervene. That is, they were not actively to resist change but were to establish the most comfortable pattern compatible with the prevailing frequency [7]. Two blocks of trials were performed for each forearm position: one block in which normal gravitational torque was present; and one block in which the weight of the hand was inverted. Within a block, trials alternated between the two prescribed patterns of coordination. A total of 12 trials (six per condition) were performed in each block, for a grand total of 48 trials. The order of presentation of the conditions was completely counterbalanced across the eight participants.

### Data Analysis

The relative phase relationship between the time of maximum (up-on-the-beat trials) or minimum (down-on-the-beat trials) displacement for each movement cycle, and the metronome beat, was derived using standard methods [8]. Abrupt changes (“transitions”) in the pattern of coordination were analysed using a partially interactive procedure. Relative phase time series were displayed via a graphical interface. Cursors were positioned at either end of regions that were obviously pre-transition or post-transition. Mean relative phase values were calculated for these regions. The relative phase value at the midpoint of the transition was also obtained. A linear regression, calculated from a 400 ms data epoch centred on this midpoint (from 200 ms before to 200 ms after), was used to derive the point in time of intersection with the pre transition mean. This point provided an index of the onset of the transition. If the departure of relative phase from its target value was followed by phase wandering, the post-transition mean was assigned a value of ±180° the target.

## Results

**Figure 1:**
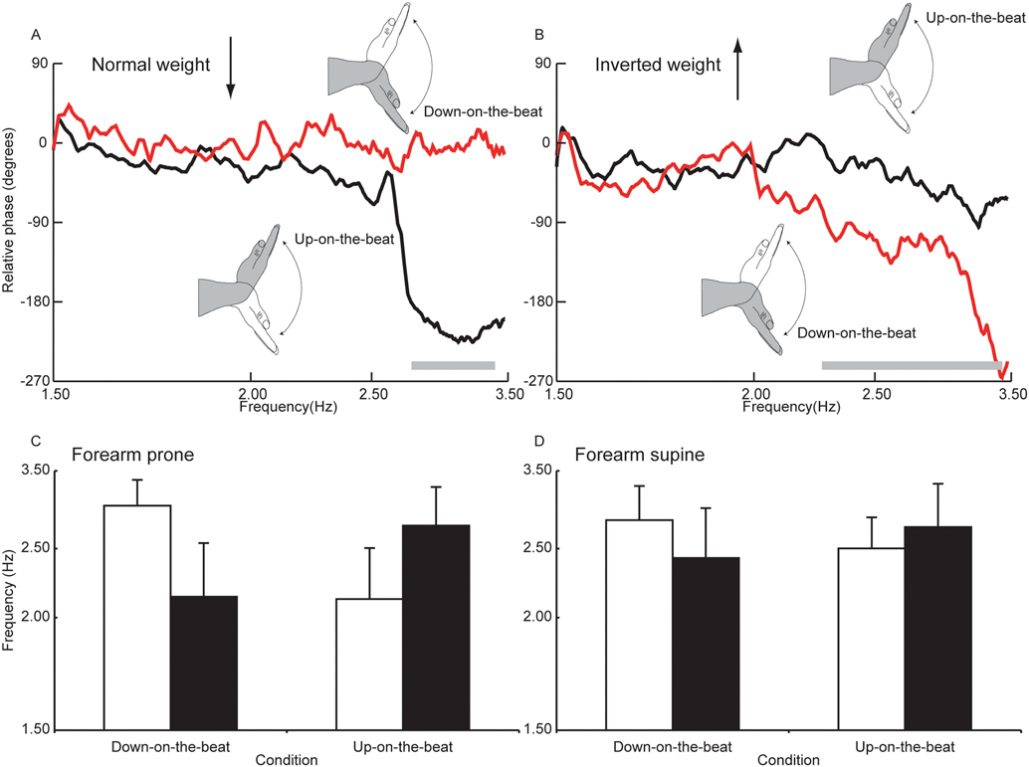
Influence of inversion of hand weight on the stability of coordination. (A) For this participant, when the gravitational torque was normal and the forearm supine, the down-on-the-beat pattern (red trace – mean of 6 trials) remained stable throughout. The up-on-the-beat pattern (black trace) became unstable initially at around 2.6 Hz, and the down-on-the-beat pattern was then adopted. (B) When the weight of the hand was inverted, the down-on-the-beat pattern was not maintained reliably beyond 2.25 Hz. In the up-on-the-beat trials no such loss of stability was exhibited. The grey horizontal bars indicate the range of frequencies across which a loss of stability occurred. (C). Mean frequency at the loss of stability (n = 8 participants) when the forearm was prone (open bars – normal weight; filled bars – weight inverted). Error bars represent means of the 95% confidence intervals obtained for the individual participants. (D) Mean frequency at the loss of stability when the forearm was supine.

Performance of the coordination task was characterised in terms of the relative phase relationship (the latency of limb motion with respect to the cycle period of the metronome) between the accentuated phase of the movement (up or down) and the beat. The principal dependent measure was the frequency at which the target pattern of coordination (relative phase≈0°) could no longer be maintained (Fig. 1A & 1B). Planned comparisons (based on ANOVA) were employed to contrast the two patterns of coordination. A three-way (forearm position [prone; supine]×gravitational torque [normal; inverted]×pattern of coordination [up-on-the-beat; down-on-the-beat]) repeated measures ANOVA design was used to compute the sums of squares and the mean squares required for the planned comparisons. An initial criterion alpha level of 0.05 was used.

In a group of trials in which the palm of the hand faced down (forearm prone: down = wrist flexion; up = wrist extension), and normal gravitational torque was present, the down-on-the-beat pattern remained stable at higher frequencies than the up-on-the-beat pattern (F(1, 7) = 106.3, p<0.01). In marked contrast, when the weight of the hand was inverted, the up-on-the-beat pattern was sustained at higher pacing frequencies than the down-on-the-beat pattern (F(1, 7) = 61.9, p<0.01) (Fig. 1C). When the movements were performed with the palm of the hand facing up (forearm supine: up = wrist flexion; down = wrist extension) an analogous pattern of results was obtained. When the weight of the hand was normal, the down-on-the-beat pattern remained more stable than the up-on-the-beat pattern (F(1, 7) = 9.82, p = 0.016), whereas when the weight of the hand was inverted, the up-on-the-beat pattern was more stable than the down-on-the-beat pattern (F(1, 7) = 11.63, p = 0.011) (Fig. 1D).

**Figure 2:**
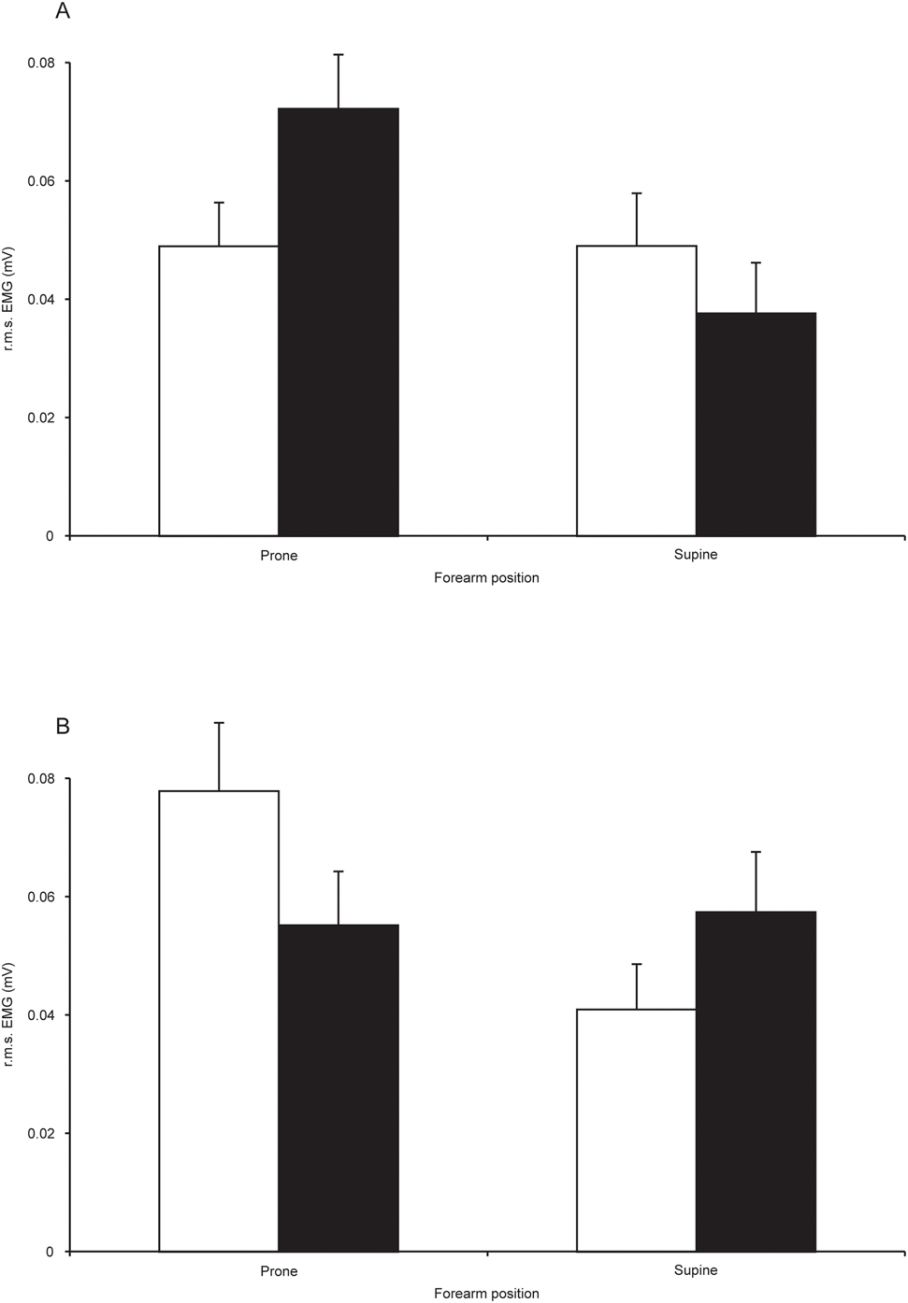
Influence of inversion of hand weight on muscle activation during performance of the coordination task. (A) Mean EMG (r.m.s.) amplitudes (n = 8 participants) of the activity recorded from the FCR muscle when the forearm was either prone or supine (open bars – normal weight; filled bars – weight inverted). Data obtained in the up-on-the-beat and down-on-the-beat trials have been combined. Error bars represent means of the 95% confidence intervals obtained for the individual participants. (B) The corresponding activity recorded from the ECR muscle.

It was verified that the muscle activity required to move the hand varied reliably in accordance with its weight. For each position of the forearm the EMG values were very similar across the normal and inverted conditions. When the forearm was prone and normal gravitational torque was present the EMG activity recorded from the flexor carpi radialis (FCR) muscle was of lower amplitude than when the weight of the hand was inverted (F(1, 7) = 243.0, p<0.01). In contrast, when the forearm was supine, EMG activity recorded from the FCR was greater when the weight of the hand was normal than when it was inverted (F(1, 7) = 58.6, p<0.01) (Fig. 2A). With respect to the extensor carpi radialis (ECR) muscle, the opposite pattern pertained. When the forearm was in a prone position, the EMG activity recorded was greater when the gravitational torque was normal than when it was inverted (F(1, 7) = 63.2, p<0.01). When the forearm was supine, the EMG obtained in ECR was of lower amplitude when the weight of the hand was normal than when it was inverted (F(1, 7) = 33.5, p<0.01) (Fig. 2B).

## Discussion

We have demonstrated that the most stable pattern of coordination is always that in which the phase of movement that is assisted by the weight of the limb is synchronised with the beat, regardless of whether that phase is up or down. The ubiquitous tendency to make a downward movement on a musical beat [9] is therefore due to the economy of action that comes from exploiting the force of gravity, rather than a structural internalisation of its orientation.

A fundamental question that arises, since the total work performed during the entire movement cycle must be equivalent regardless of whether the weight of the hand is normal or inverted, is why it is critical in terms of enhancing the stability of coordination that the phase of the action that is synchronised with the environmental stimulus (i.e. the metronome) is assisted by gravity. In order to seek an explanation for this phenomenon it is necessary to consider the predominantly linear correspondence that exists between the magnitudes of the force and time components of muscle-generated impulses and their respective variances [10].

By definition, sensorimotor coordination requires that completion of the accentuated phase of the movement cycle occur at the same time as the stimulus. Accurate and stable performance is necessarily characterised by compression of both spatial and temporal variability at this single point in the movement cycle [11], [12]. The consequences of the economy of action that comes from exploiting the force of gravity can therefore be readily appreciated. Greater force must be produced during movement phases that are opposed by gravity than during phases that are assisted by gravity. There will be a corresponding elevation in the variance of both the force and time components of the muscle-generated impulse during gravity opposed phases, and a consequential increase in the spatial and temporal variability of the resulting movement. This translates to a reduced stability of coordination. It appears to be a general organising principle that in the presence of noise that scales with the magnitude of the motor output, the CNS selects movement trajectories that minimise the variance of the terminal position of the limb [13]. As the muscle-generated impulse is smaller for the phase of the movement cycle assisted by the weight of the limb, adopting a pattern in which this phase is coordinated with the beat compresses spatial and temporal variability at the point of synchronisation.

The present findings are consistent with a larger body of research indicating that sensorimotor coordination is particularly stable when the relative force requirements are low. When the strength of muscles that generate one phase (e.g. extension) of a rhythmic movement cycle is promoted by resistance training, the stability of a pattern of coordination that requires this phase to be accentuated is correspondingly increased [14]. It has also been shown that temporary changes in force generating capacity brought about by alterations of limb posture, that arise from the force-length relationship of skeletal muscle [15], have similar systematic effects upon the stability of synchronisation tasks [8], [16], [17], as does the introduction of a viscous load that opposes one phase of the movement cycle [18].

It is known that an elevation in the rate at which auditory stimuli are presented is accompanied by increases in fMRI derived signal in the lateral temporal lobes [19]. In addition, qualitative changes in auditory network activity registered by magnetoencephalography [20] have been reported in association with involuntary transitions from syncopated (between the beats) to synchronised (on the beats) patterns of sensorimotor coordination. Nonetheless, recent observations that the functional coupling between primary auditory areas and key elements of the cortical motor network remains stable for a given mode of coordination, with elevation of the rate of movement or auditory presentation [21], suggest that the transitions in behaviour obtained in the present study cannot be accounted for on this basis. Rather, the key neural mechanisms are likely to be those that mediate the relationship between the magnitude of the muscle-generated impulse necessary to perform an action and the consequential spatial and temporal variability of that action [22].

The cortical representations of muscles overlap broadly (e.g. [23], [24]), and dense connectivity exists between multiple frontal motor areas [25]. Even activity in highly localised regions of the cortical motor cortex has the potential to influence a wide variety of muscle synergies [26]. Factors such as elevations in the rate of movement [27]–[29], or the requirement to move against rather than with the force of gravity, which necessitate greater motor output, thus have the capacity to increase the interference that occurs between the cortical representations of the focal muscles required in a movement task and of those that do not contribute directly to the desired action [30], [31]. This interference finds expression in the variance of the force and time components of muscle-generated impulses, and ultimately as elevations in the spatial and temporal variability of movement.

The transitions observed in the present study reflect the expression of a general organising principle based on economy of action. Other factors being equal, the CNS preferentially adopts patterns of sensorimotor coordination, whereby the accentuated phase of the movement cycle is that which has the lowest force requirements, thus ensuring that the variance of the terminal position of the limb is minimised at the point of synchronisation. The pervasive tendency in circumstances of normal gravity to adopt patterns of movement that ensure that the end of the downward phase of our gestures coincide with the beat of the music is entirely consistent with this principle. As the muscle-generated force impulse is smaller during the accentuated phase than during the upward phase, moving down on the beat achieves the greatest possible stability of sensorimotor coordination.
